# Ga(III) Nanoparticles Inhibit Growth of both Mycobacterium tuberculosis and HIV and Release of Interleukin-6 (IL-6) and IL-8 in Coinfected Macrophages

**DOI:** 10.1128/AAC.02505-16

**Published:** 2017-03-24

**Authors:** Seoung-ryoung Choi, Bradley E. Britigan, Prabagaran Narayanasamy

**Affiliations:** a Department of Pathology and Microbiology, College of Medicine, University of Nebraska Medical Center, Omaha, Nebraska, USA; b Department of Internal Medicine, College of Medicine, University of Nebraska Medical Center, Omaha, Nebraska, USA; c Research Service, VA Medical Center Nebraska-Western Iowa, Omaha, Nebraska, USA

**Keywords:** IL-6, IL-8, Mycobacterium tuberculosis, human immunodeficiency virus, nanoparticle

## Abstract

Treatment of individuals coinfected with human immunodeficiency virus (HIV) type 1 and Mycobacterium tuberculosis is challenging due to the prolonged treatment requirements, drug toxicity, and emergence of drug resistance. Mononuclear phagocytes (MP; macrophages) are one of the natural reservoirs for both HIV and M. tuberculosis. Here, the treatment of HIV and M. tuberculosis coinfection was studied by preloading human macrophages with MP-targeted gallium (Ga) nanoparticles to limit subsequent simultaneous infection with both HIV and M. tuberculosis. Ga nanoparticles provided sustained drug release for 15 days and significantly inhibited the replication of both HIV and M. tuberculosis. Addition of Ga nanoparticles to MP already infected with M. tuberculosis or HIV resulted in a significant decrease in the magnitude of these infections, but the magnitude was less than that achieved with nanoparticle preloading of the MP. In addition, macrophages that were coinfected with HIV and M. tuberculosis and that were loaded with Ga nanoparticles reduced the levels of interleukin-6 (IL-6) and IL-8 secretion for up to 15 days after drug loading. Ga nanoparticles also reduced the levels of IL-6 and IL-8 secretion by ionomycin- and lipopolysaccharide-induced macrophages, likely by modulating the IκB kinase-β/NF-κB pathway. Delivery of Ga nanoparticles to macrophages is a potent long-acting approach for suppressing HIV and M. tuberculosis coinfection of macrophages *in vitro* and sets the stage for the development of new approaches to the treatment of these important infections.

## INTRODUCTION

Human immunodeficiency virus (HIV) type 1 (HIV-1) and Mycobacterium tuberculosis are two major infectious agents that cause high rates of mortality worldwide. M. tuberculosis, the causative agent of tuberculosis (TB), is one of the leading causes of death in the world. In 2015, the World Health Organization (WHO) estimated that 9 million people developed TB and 1.5 million died from it. Of the 9 million people who developed TB, 13% were also HIV positive (1). Furthermore, HIV-associated M. tuberculosis infections and deaths from HIV-associated M. tuberculosis infections are increasing in frequency throughout the world (2).

Numerous challenges to the treatment of patients coinfected with HIV and M. tuberculosis have emerged. Multidrug-resistant M. tuberculosis and extensively drug-resistant M. tuberculosis strains are a growing problem and often occur in the setting of coexisting HIV infection (3). In addition, treatment of patients with TB in association with HIV infection requires the use of prolonged multidrug treatment regimens that interact with some antiretroviral drugs, increasing the potential for drug toxicity (4). Thus, an urgent need exists for simplified, long-acting, and effective regimens to treat HIV and M. tuberculosis coinfection (5).

In the pathogenesis of M. tuberculosis infection, the bacillus invades and multiplies intracellularly within monocytes and macrophages. The primary initial target of the bacillus is alveolar macrophages. There the bacilli replicate until their growth is restricted by activation of the macrophages by gamma interferon (IFN-γ) and other factors released from T cells (6, 7). The infection is contained within the lungs by the formation of granulomas comprised of M. tuberculosis-infected macrophages, dendritic cells, and T cells.

After years of dormancy, M. tuberculosis can begin to multiply and cause reactivation disease if the host's immune function decreases. HIV infection amplifies the risk for developing active TB by decreasing T cell-mediated immunity, resulting in the reactivation of latent M. tuberculosis infection. HIV infection accelerates the rupture of granules, releasing active M. tuberculosis bacteria that are transported by dendritic cells to the lymph nodes (6).

Both HIV and M. tuberculosis target and replicate in macrophages, in turn weakening human immunological functions. The intracellular replication of M. tuberculosis or HIV eventually leads to cell death and the extracellular release of the pathogen (8). Maintenance of the active infection within the host requires that the infecting pathogen have the ability to continually establish infection in newly arriving susceptible cell types. Failure to do so would be expected to result in the termination of active infection.

Macrophages are located in various tissues and play important roles in immunity by engulfing pathogens, eliminating apoptotic cells, and recycling nutrients. Upon encountering bacteria and other inflammatory stimuli, macrophages secrete proinflammatory cytokines like tumor necrosis factor (TNF), interleukin-1 (IL-1), IL-6, IL-8, and IL-12. Some cytokines are potent pyrogens that activate and recruit other cells to sites of microbial invasion/infection. The formation of granulomas is a pathological hallmark of the host response to M. tuberculosis infection. Several cytokines have been identified in granulomas from patients with M. bovis BCG infection. Here, IL-6 is involved in the pathological functioning of M. tuberculosis infection (9). IL-8 also controls granuloma formation, which follows leukocyte influx on M. tuberculosis infection. High levels of IL-8 are observed in M. tuberculosis-infected human tissue, plasma, pleural fluid, and bronchoalveolar lavage fluid. In addition, in *in vivo* studies, pretreatment with anti-IL-8 inhibited mycobacterial granuloma formation (10). Cytokines also play a pivotal role in maintaining granulomas with the aid of CD4^+^ T cells (11). However, some cytokines enhance HIV replication in macrophages (12).

To survive in humans and to be pathogenic, M. tuberculosis has to be able to acquire critical nutrients. Among these nutrients is iron (Fe), whose acquisition is required for the survival of mycobacteria residing in human macrophages (13–15). Iron's ability to undergo redox cycling between the ferrous (Fe^2+^) and ferric (Fe^3+^) oxidation states allows it to function as an electron transporter in many enzymatic systems, including those involved in DNA replication and cellular energy production (16). Thus, blocking of Fe acquisition by M. tuberculosis is a potential way to reduce the growth of M. tuberculosis within macrophages (17).

Gallium (Ga) is a trivalent cationic element with many features that are similar to those of Fe, making it largely indistinguishable from Fe to many biological systems. Ga can interact with biologically important proteins that are involved in Fe metabolism, interfering with both Fe acquisition mechanisms and the function of Fe-dependent enzymes, including catalases, Fe superoxide dismutase, and ribonucleotide reductase. Insertion of Ga(III) into the active site of these normally Fe-centered enzymes renders them inactive, as, in contrast to Fe, Ga(III) cannot be reduced to Ga(II) in biological systems (18). Ga(NO_3_)_3_ is an FDA-approved treatment for hypercalcemia associated with cancer and concentrates in activated macrophages (19). We previously demonstrated that Ga disrupts Fe acquisition by mycobacteria and iron metabolism, leading to inhibition of the growth of these and other intracellular pathogens (14, 20–23).

Antimicrobial nanoparticles (NP) that target macrophages have great potential advantages for drug delivery and clinical efficacy in the inhibition of intracellular pathogens. Such macrophage-targeted nanoparticles have been developed to inhibit virus replication (24, 25). Monocyte-derived macrophage (MDM)-targeting nanoparticles containing conventional antiretroviral therapy have been reported to lengthen the activities and efficacies of existing therapies against HIV-1 infection with less toxicity (26, 27).

**FIG 1:**
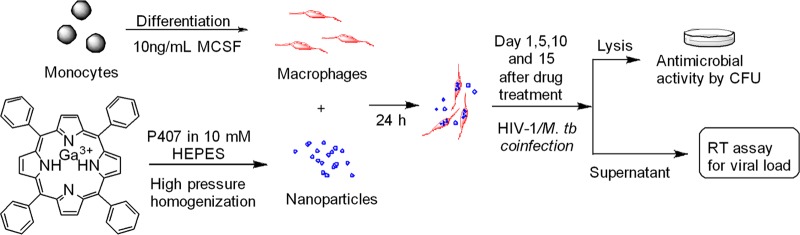
Scheme for testing antimicrobial activities of gallium(III) in M. tuberculosis (*M. tb*)- and HIV-infected MDMs.

In a recent study, we showed that a long-acting Ga nanoparticle (GaNP) formulation inhibited the growth of both HIV and M. smegmatis residing within MDMs by releasing Ga(III) over 15 days after the cells were loaded with the drug (8). No significant cytotoxicity resulting from these Ga nanoparticles was observed (8). The results encouraged us to explore the potential of Ga nanoparticles for the treatment of HIV and M. tuberculosis coinfection (Fig. 1). Furthermore, we studied the impact of these Ga nanoparticles on cytokine release by macrophages infected with M. tuberculosis and HIV-1.

## RESULTS AND DISCUSSION

**FIG 2:**
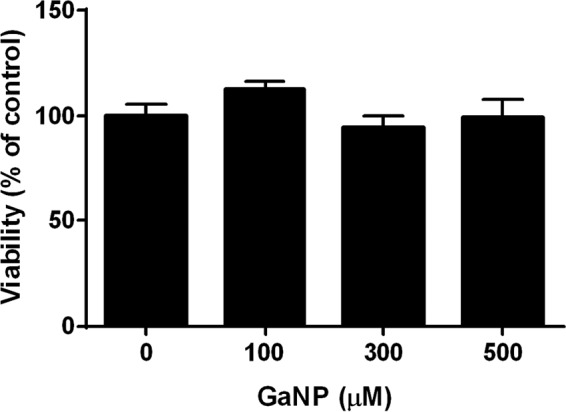
Toxicity of GaNP against THP-1 macrophages. A total of 0.75 × 10^6^ cells/well were seeded into each well of a 24-well plate. The wells were treated with various concentrations of GaNP for 24 h, and then the wells were thoroughly washed with PBS buffer. The GaNP-treated macrophages were further incubated for an additional 15 days, with the medium being changed every 2 days, and viability was determined using a resazurin reduction assay.

In order to optimize macrophage-utilized drug delivery, various physical properties of Ga nanoparticles were tested to increase the encapsulating potential, prolong the duration of drug release, and minimize cytotoxicity. This resulted in the development of gallium nanoparticles (GaNP) using water-insoluble gallium(III) *meso*-tetraphenylporphyrin (GaTP) and P407 pluronic polymer and a high-pressure homogenization technique (8). Excellent drug loading (up to 48%) was achieved, and the scanning electron microscopy (SEM) image showed a rod-like shape. The potential for cytotoxicity of various concentrations (25 to 500 μM) of GaNP on THP-1 macrophages was assessed for up to 15 days after loading of the cells with the nanoparticles (Fig. 2; see also Fig. S1 in the supplemental material). No toxicity, as assessed by resazurin reduction, was detected. The uptake of Ga nanoparticles by MDMs was observed and led to sustained Ga release for 15 days (8).

The pathogenesis of infection with M. tuberculosis and HIV requires the ongoing infection of uninfected host cells migrating to the site of infection. Interruption of this process by increasing the resistance of uninfected cells to infection should terminate infection. Therefore, we assessed the susceptibility of MDMs that had been pretreated with Ga nanoparticles 1 day, 5 days, 10 days, and 15 days earlier to infection with M. tuberculosis and/or HIV compared to that of untreated MDMs. These studies were designed to test the duration of protective efficacy of the gallium nanoparticle against the two pathogens. Defining the duration of Ga drug availability after loading of the macrophages with drug is also critical to a potential strategy of using drug-loaded macrophages to deliver Ga drugs to sites of infection.

### GaNP decreases the growth of M. tuberculosis in MDMs and THP-1 macrophages.

**FIG 3:**
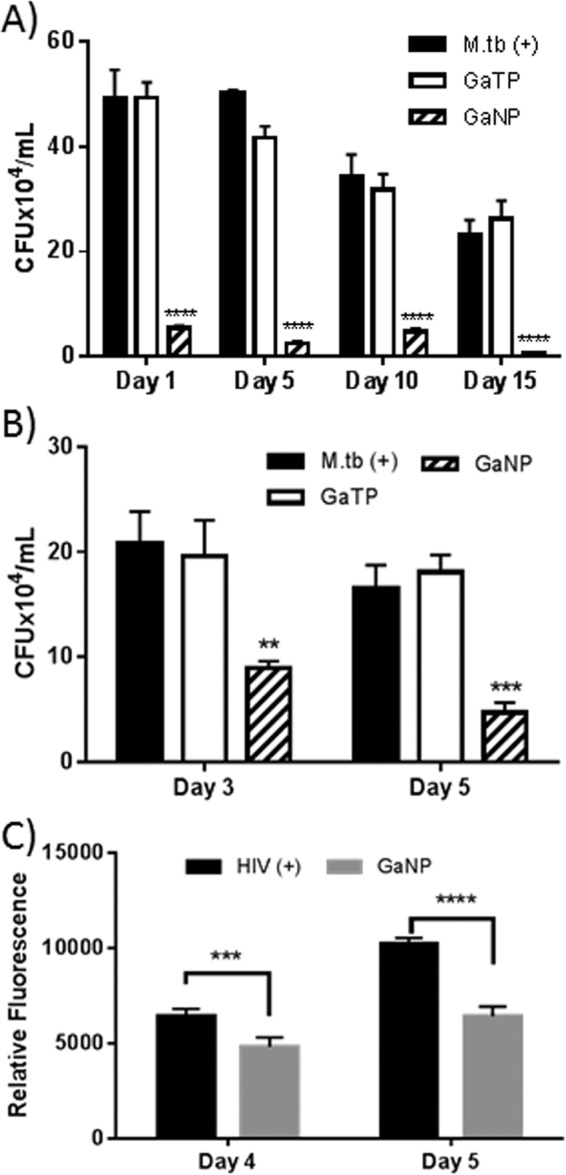
Antimicrobial activities of gallium(III). (A) MDMs were incubated with GaTP or GaNP for 24 h, following which the cultures were washed free of extracellular drug. At days 1, 5, 10, and 15 following drug treatment, these MDMs, as well as control MDMs that had not been exposed to drug, were then infected with M. tuberculosis (H37Ra; MOI = 1) for 4 h, and then the M. tuberculosis bacteria were allowed to grow for 2 days. (B, C) M. tuberculosis (B) and HIV-1 (C) growth inhibition in infected THP-1 macrophages by Ga nanoparticles. THP-1 macrophages were infected with H37Ra strain (MOI = 1) or HIV-1 (MOI = 0.01) and then incubated with GaNP for 24 h. Growth inhibition was monitored over time by determining the number of CFU for M. tuberculosis and by an RT assay for HIV-1. Ga was used in the form of GaTP (300 μM) or GaNP (300 μM). Data represent the mean ± standard error of the mean for triplicate wells (*n* = 3). Statistically significant differences were determined using Student's *t* test. **, *P* < 0.01 compared with the non-drug-treated control; ***, *P* < 0.001 compared with the non-drug-treated control; ****, *P* < 0.0001 compared with the non-drug-treated control.

We assessed the antimycobacterial activities of GaTP and GaNP on human MDMs infected with M. tuberculosis. MDMs were incubated with GaTP or GaNP for 24 h, washed, and then placed back into culture. At defined time points after exposure to GaTP or GaNP (1, 5, 10, or 15 days), the cells were incubated with M. tuberculosis (H37Ra) for 4 h, washed free of extracellular bacteria, and then placed back in culture. After an additional 2 days of incubation, the MDMs were lysed and the number of M. tuberculosis CFU was determined. Even only 1 day after drug loading, the free drug GaTP showed a minimal inhibitory effect on M. tuberculosis, with the number of CFU being similar to that obtained with untreated control MDMs (Fig. 3A). In contrast, GaNP exhibited a significant inhibition (more than 10-fold compared to that of GaTP) of the growth of M. tuberculosis, even when infection of the MDMs occurred as long as 15 days after drug loading. The numbers of CFU detected in drug-treated and control MDMs infected at 10 days after drug treatment and beyond were less than the numbers detected in MDMs infected with M. tuberculosis following drug exposure (Fig. 3A). The results obtained with GaNP, which were consistent with our data previously obtained with M. smegmatis (8), suggest that the incorporation of Ga into nanoparticles delivered into MDMs leads to the sustained release of Ga into the intracellular compartment at a level sufficient to inhibit the growth of M. tuberculosis up to 15 days after loading of the MDM with drug.

Iron (Fe) is a nutrient critical for the survival of mycobacteria residing in human macrophages (13). In earlier studies, gallium nitrate was shown to significantly inhibit intracellular mycobacterial growth. This appeared to occur via disruption of bacterial iron metabolism, as addition of exogenous iron to the medium decreased the antimicrobial effect of gallium nitrate (14). We compared the activity of gallium nitrate with that of GaNP. At 5 days after incubation of the macrophages with the two drugs, both gallium nitrate and GaNP produced similar reductions in M. tuberculosis growth (Fig. S3). However, GaNP continued to limit bacterial growth for 15 days after loading of the macrophage with drug (Fig. 3A). To provide insight into the role of iron limitation in GaNP-mediated inhibition of M. tuberculosis growth in MDMs, GaNP was added to THP-1 macrophages in the presence and absence of FeNO_3_, followed by M. tuberculosis infection. Interestingly, we did not see any difference in bacterial growth (Fig. S2). Some possible explanations for the observed result are that it might have been due to the higher intracellular concentrations of Ga achieved with GaNP, differences in the timing of M. tuberculosis infection and gallium treatment compared to those used in the prior study, and the effect of the nanoparticle. A detailed mechanistic study will need to be carried out to determine the exact explanation.

### HIV growth increases in the presence of M. tuberculosis and HIV coinfection of MDMs.

**FIG 4:**
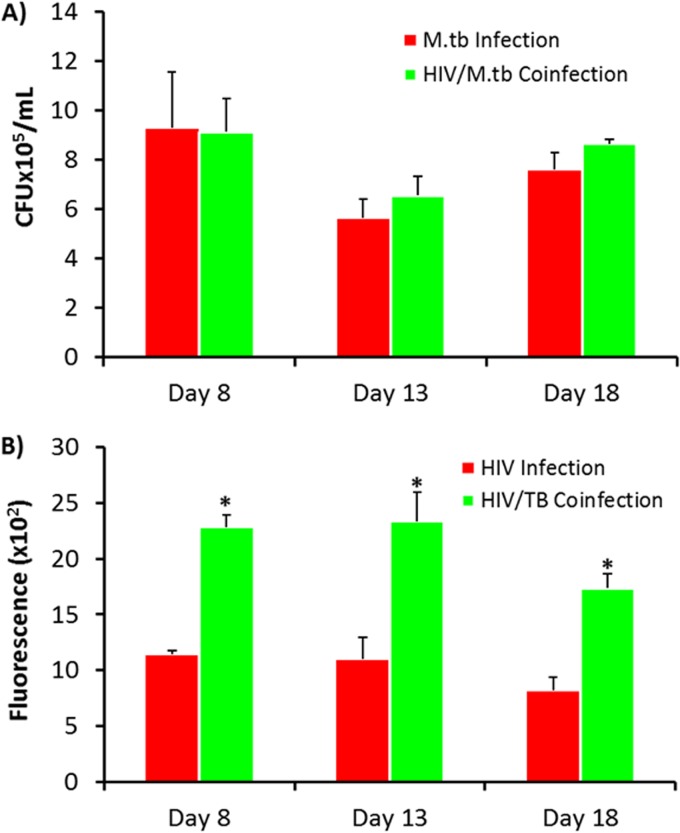
Quantitation of M. tuberculosis growth and HIV replication in MDMs coinfected with HIV-1 and M. tuberculosis. (A) M. tuberculosis growth was determined by counting of the number of CFU. (B) HIV-1 in human MDMs was quantitated by a reverse transcriptase assay. MDMs were infected with HIV-1 (MOI = 0.01) or M. tuberculosis (H37Ra; MOI = 1) or coinfected with HIV-1 and M. tuberculosis at days 8, 13, and 18 following differentiation. The coinfected MDMs and HIV-infected MDMs were further incubated for 11 days, and the medium was changed every 48 h. Supernatants from cells coinfected with HIV-1 and M. tuberculosis were harvested at day 11 for analysis of RT activity. Data represent the mean ± standard error of the mean for triplicate wells. Statistically significant differences were determined using Student's *t* test. *, *P* < 0.01 compared with HIV-infected cells in panel B.

In preparation for extension of the studies of GaNP to MDMs that were coinfected with M. tuberculosis and HIV, we determined the effect of coinfection of the MDMs on the rates of M. tuberculosis and HIV-1 growth. The growth of M. tuberculosis bacteria residing within infected MDMs *in vitro*, as assessed by determination of the number of CFU, was not significantly different in the presence of HIV over 2 days (Fig. 4A). This lack of an apparent difference may be due to the fact that M. tuberculosis grows slowly, and here, our determinations were made after only 2 days, possibly masking a difference in growth at a later stage of coinfection. In contrast, *in vitro* coinfection of MDMs with M. tuberculosis (H37Ra) and HIV-1 increased the levels of HIV-1 up to 2-fold, as assessed by a reverse transcriptase (RT) assay, over the course of 11 days in culture (Fig. 4B). The viability of MDMs was decreased by infection with M. tuberculosis and HIV, but the cells survived up to 15 days, as viewed by inverted phase microscopy.

### Ga(III) nanoparticles inhibited the growth of M. tuberculosis and the replication of HIV-1 residing in MDMs.

**FIG 5:**
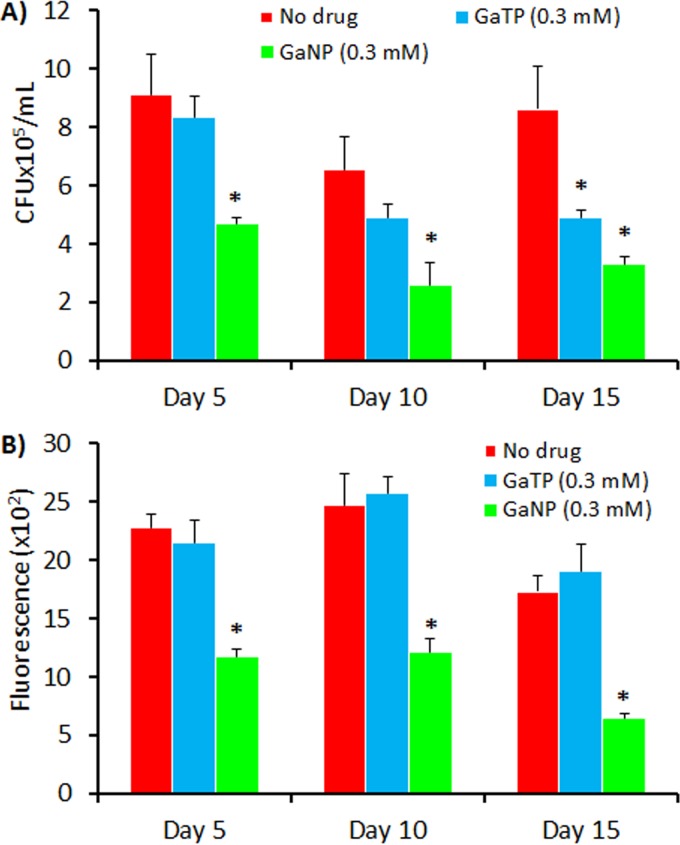
Antimicrobial activities of gallium(III) in MDMs coinfected with HIV-1 and M. tuberculosis. Antimicrobial activities were determined by counting of the number of CFU for M. tuberculosis (A) and an RT assay for HIV-1 (B). GaTP (300 μM)- and GaNP (300 μM)-treated MDMs were infected with both HIV-1 and M. tuberculosis (H37Ra) at days 5, 10, and 15 following treatment of the MDMs with drug. Data represent the mean ± standard error of the mean for 3 or 6 wells. Statistically significant differences were determined using Student's *t* test. *, *P* < 0.01 compared with the non-drug-treated control.

As shown in Fig. 3, GaNP treatment of MDMs resulted in the decreased growth of M. tuberculosis when infection was initiated up to 15 days after drug loading occurred. HIV-1 replication requires Fe-dependent protein ribonucleotide reductase, NF-κB, CDK9, elongation factor F5a (elF5a), and ABCE1, and recent work from our labs has shown the ability of Ga to inhibit HIV replication in MDMs (8). Therefore, we investigated the antimicrobial activities of GaNP on MDMs coinfected with HIV-1 and M. tuberculosis (Fig. 5).

Consistent with the results from the experiments whose results are shown in Fig. 3, GaNP showed long-acting inhibitory activity against M. tuberculosis; i.e., significant M. tuberculosis growth inhibition was observed up to 15 days after incubation of the MDMs with GaNP.

Consistent with the earlier data from our labs (8), MDMs that were treated with GaNP and that were coinfected with M. tuberculosis and HIV-1 exhibited significantly reduced levels of replication of HIV (2-fold) compared to that for nontreated MDMs. In contrast, free drug (GaTP) did not have a significant inhibitory effect (Fig. 5B). As with M. tuberculosis, HIV-1 growth inhibition persisted for 15 days after loading of the MDMs with GaNP (Fig. 5B). Interestingly, GaNP reduced the growth of M. tuberculosis residing in MDMs by 10-fold compared to that for nontreated MDMs, while a 2-fold decrease in the growth of M. tuberculosis in coinfected MDMs was observed. This finding may indicate that M. tuberculosis and HIV compete for Ga uptake in an environment with limited amounts of Ga, resulting in less GaNP-induced killing of M. tuberculosis.

The effect of GaNP administered after THP-1 macrophages were infected with M. tuberculosis and/or HIV was also investigated. GaNP treatment after infection resulted in a significant reduction of bacterial growth, with growth being inhibited by 61% and 70% at days 3 and 5, respectively, compared to that for their positive controls (Fig. 3B). GaNP treatment after infection also inhibited HIV growth by 25% and 37% at days 4 and 5, respectively (Fig. 3C). The magnitude of the effect of GaNP on the M. tuberculosis load in the macrophages appeared to be greater with GaNP treatment before infection (Fig. 3A) than treatment after infection (Fig. 3B and Fig. 3C). Inherently, GaNP treatment before infection appeared to decrease the initial levels of HIV and M. tuberculosis infection of the cells compared to that for the control (Fig. 3). Thus, it seems possible that GaNP is able to block both the initial infection and the further replication of M. tuberculosis and HIV but is unable to decrease the burden of infection of a cell beyond that present when GaNP treatment begins.

### Secretion of cytokines by MDMs coinfected with HIV-1 and M. tuberculosis.

Once a host is exposed to M. tuberculosis, the host immune defense response is initiated by the production of a variety of molecules, including cytokines and chemokines, and by the development of a T cell-mediated immune response (28). These T cells produce gamma interferon (IFN-γ), which plays an important role in activating macrophages to produce reactive oxygen and nitric oxide species and release other cytokines and chemokines (6). IFN-γ, tumor necrosis factor alpha (TNF-α), and IL-12 appear to be key cytokines in controlling M. tuberculosis infection in mice and humans (29, 30).

In parallel to our initial studies examining the effect of macrophage loading with GaNP on the growth of M. tuberculosis (Fig. 3), we also determined the effect of GaNP loading on the release of IL-6 and IL-8 from M. tuberculosis-infected MDMs. Interestingly, GaNP reduced the amount of IL-6 and IL-8 released from M. tuberculosis-infected MDMs (see the supplemental material) in parallel with reducing the number of M. tuberculosis CFU (Fig. 3).

**FIG 6:**
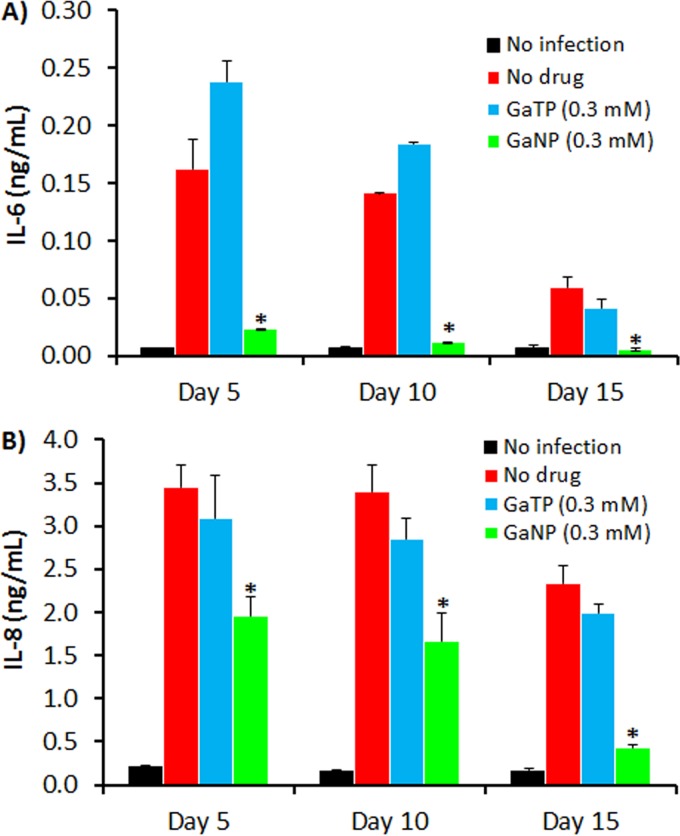
Analysis of cytokines present in supernatants from macrophages coinfected with HIV-1 and M. tuberculosis. (A) IL-6; (B) IL-8. MDMs were coinfected with HIV-1 and M. tuberculosis (H37Ra) at days 5, 10, and 15 following MDM incubation with GaTP (300 μM) and GaNP (300 μM). On day 11 after infection, supernatants were analyzed for the presence of cytokines released from infected MDMs. The data represent the mean ± standard error of the mean for 3 or 6 wells. Statistically significant differences were determined using Student's *t* test. *, *P* < 0.05 compared with the positive controls coinfected with HIV-1 and M. tuberculosis.

Coinfection of macrophages with M. tuberculosis and HIV may also alter the profile of cytokines produced by macrophages. In order to investigate this possibility, the cytokine profiles of culture supernatants from MDMs coinfected with HIV and M. tuberculosis were analyzed using a Luminex system. As seen in Fig. 6, of 10 cytokines assessed, two major interleukins (IL-6 and IL-8) were detected in large quantities, with trace amounts of IL-1β, TNF-α, IFN-γ, macrophage colony-stimulating factor (M-CSF), and IL-4 being detected (see the supplemental material). No significant amount of TNF-α (<3 pg/ml) was detected, which is in good agreement with the findings of other studies of HIV and M. tuberculosis coinfection (31). Also, the TNF-α released by macrophages facilitates macrophage apoptosis in response to M. tuberculosis (32–34). Patel et al. (31) also observed that HIV infection reduced macrophage apoptosis in response to M. tuberculosis due to the reduced production of TNF-α.

It has been suggested that IFN-γ and TNF-α can serve as excellent biomarkers for the diagnosis of TB (35). Recently, IL-6 was also suggested to be a potent biomarker in mycobacterial (M. tuberculosis H37Rv and H37Ra, M. smegmatis) infections of mouse peritoneal macrophages (36). Although that study (36) was conducted with murine macrophages *in vitro*, the high level of IL-6 release is consistent with the findings of a study with human macrophages (37). As shown in Fig. 6A, the amount of IL-6 found in culture supernatants from MDMs coinfected with HIV and M. tuberculosis was significantly larger than the amount found in culture supernatants from uninfected MDMs, indicating that HIV infection does not influence IL-6 release by M. tuberculosis-infected MDMs, supporting the potential for the use of IL-6 as a biomarker in mycobacterial infection of HIV-coinfected patients.

Interestingly, the level of IL-6 production by MDMs loaded with GaNP for 5 and 10 days prior to infection with M. tuberculosis and HIV was 6-fold less than that by control cells that were not treated with Ga (Fig. 6A). When infection occurred at day 15 after GaNP loading, the levels of IL-6 in treated or nontreated MDMs were lower than those in MDMs infected at days 5 and 10 after drug loading (Fig. 6A). This was likely at least in part due to a decrease in the viability of macrophages with a longer time in culture. However, GaNP-treated MDMs still showed a 6-fold lower level of IL-6 release than the non-GaNP-treated control MDMs (Fig. 6A). In contrast to the results obtained with GaNP, MDMs that received free drug (GaTP) (Fig. 6A) or nanoparticles not containing GaTP (not shown) exhibited IL-6 responses similar to those of the non-Ga-treated control cells.

We also found that the level of production of IL-8 by MDMs coinfected with HIV and M. tuberculosis was 20-fold higher than that by uninfected control MDMs, regardless of how many days they were in culture prior to infection (Fig. 6B). IL-8 is a proinflammatory chemokine produced by monocytes, macrophages, endothelial cells, and other types of cells. Its main role is to recruit neutrophils, as well as T lymphocytes and monocytes, to sites of infection (38). The main sources of IL-8 are monocytes and macrophages infected with pathogens, including mycobacteria (39). Although higher levels of IL-8 along with higher levels of IL-6 are found in the pleural fluid of patients infected with M. tuberculosis or coinfected with HIV and M. tuberculosis, there are no significant differences in the patterns or the levels of cytokines between the two groups (40). Recently, Krupa et al. suggested that IL-8 can bind to M. tuberculosis and that this association may enhance the immune response in patients with TB (41). The results of these studies are in good agreement with those of our *in vitro* study of IL-6 and IL-8 secretion in response to M. tuberculosis and HIV infection of MDMs.

Similar to the findings for IL-6, GaNP treatment also significantly reduced the level of secretion of IL-8 by MDMs coinfected with HIV and M. tuberculosis (Fig. 6B). This effect was maximal in cells that were infected at day 15 after they were loaded with GaNP (Fig. 6B), where the level of IL-8 production was equal to that by uninfected MDMs.

**FIG 7:**
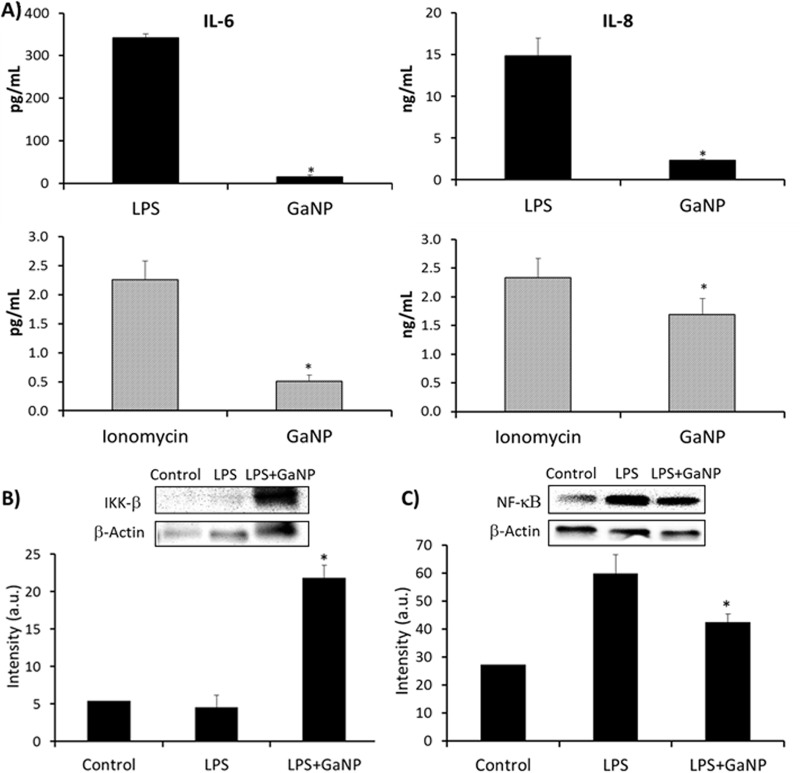
(A) Release of IL-6 and IL-8 by MDMs in the presence of LPS (10 μg/ml) or ionomycin (1 μM) compared with that by GaNP-treated MDMs exposed to the same agents. Preloading with GaNP significantly reduced the levels of IL-6 (*, *P* < 0.01) and IL-8 (*, *P* < 0.05) generation induced by LPS and ionomycin. (B) Western blot analysis of IKK-β. The overexpression of IKK-β was observed in the presence of GaNP. (C) Western blot analysis of NF-κB. The downregulation of NF-κB was observed in the presence of GaNP, confirming the relation with IKK-β. Statistically significant differences were determined using Student's *t* test (*n* = 3). *, *P* < 0.05 compared with non-drug-treated MDMs. a.u., arbitrary units.

The data presented above show that GaNP decreases M. tuberculosis- and HIV-induced IL-6 and IL-8 production, which could occur by inhibiting M. tuberculosis and/or HIV replication in MDMs. However, it is also possible that GaNP has an effect on the ability of MDMs to produce IL-6 and/or IL-8 by altering an MDM signaling pathway needed for the production of these cytokines. Therefore, the effect of GaNP on the release of IL-6 and IL-8 by MDMs in response to lipopolysaccharide (LPS; 10 μg/ml), ionomycin (1 μM), and M-CSF (10 ng/ml) (see the supplemental material) was investigated. Interestingly, GaNP was able to significantly inhibit MDM IL-6 and IL-8 production in response to LPS and ionomycin (Fig. 7A). We also observed changes in the regulation of IκB kinase-β (IKK-β)/NF-κB by GaNP (Fig. 7B). By Western blotting, we observed the increased expression of IKK-β in the presence of GaNP. In general, IKK-β is an NF-κB inhibitor, and accordingly, we observed the downregulation of NF-κB by Western blotting. By Western blotting, we did not see any changes in phospho-Akt or phospho-p38 expression in the presence of GaNP (Fig. S10 and S11). These results are consistent with the possibility that GaNP decreases IL-6 and IL-8 levels by targeting the IKK-β signaling pathway.

In general, IL-6 and IL-8 production appear to have a negative effect on the host defense against M. tuberculosis (9, 42, 43). IL-6 promotes M. tuberculosis growth and inhibits the production of TNF-α and IL-1β, which are needed for maximal killing of M. tuberculosis (9, 43). Similarly, IL-8 increases the levels of inflammation and granuloma formation induced by M. tuberculosis (42) Therefore, the GaNP-mediated inhibition of both IL-6 and IL-8 could further enhance the host defense against M. tuberculosis beyond the drug's ability to inhibit the growth of the organism through the disruption of Fe metabolism. GaNP is able to reduce M. tuberculosis growth for a longer period independently of HIV and when it is present as a coinfection with HIV. In addition, it is also able to regulate the secretion of both IL-6 and IL-8 by MDMs, likely by interfering in the IKK-β/NF-κB cell signaling pathway.

In summary, the effect of GaNP loading of human MDMs on *in vitro* coinfection of these macrophages with HIV-1 and M. tuberculosis was studied. Up to 15 days after MDMs were loaded with the drug, GaNP was able to reduce the growth of both M. tuberculosis and HIV-1. Given what is known to date about the mechanism of action of Ga, this is most likely due to the disruption of iron metabolism critical to pathogen growth/survival, although this could not be confirmed in the present work.

*In vitro*, MDMs coinfected with HIV-1 and M. tuberculosis exhibited increased levels of production of IL-6 and IL-8 but produced negligible amounts of TNF relative to uninfected macrophages. GaNP-treated MDMs showed reduced levels of IL-6 and IL-8 production. This could have been due to the inhibition of M. tuberculosis and HIV infection and/or a GaNP-mediated modification of a macrophage signaling pathway(s) that resulted in the production of these two cytokines.

Development of Ga(III)-based drugs that target human macrophages may be a potential approach for the treatment of coinfection with HIV-1 and M. tuberculosis and the reduction of IL-6 and IL-8 generation. A similar approach could allow targeting of organisms present in other cellular reservoirs. In addition, improvement of drug loading, cell targeting, encapsulation efficiency, and sustained drug release will also be important for the optimization of treatment.

## MATERIALS AND METHODS

### Ethics statement.

The human cell samples were purchased from and provided in an anonymized fashion by the University of Nebraska Medical Center (UNMC) Department of Pharmacology and Experimental Neuroscience cell core facility. Experiments with human samples were performed in full compliance with the regulations of the National Institutes of Health. All participants involved in this study provided informed written consent. The methods were carried out in accordance with approved UNMC ethical guidelines. The UNMC Institutional Review Board (IRB) approved all experimental protocols (approval number 162-93-FB, Leukapheresis of normal donors for use in studies of disease pathogenesis and therapy).

### Preparation and characterization of gallium nanoparticles.

Gallium(III) *meso*-tetraphenylporphyrine chloride (GaTP) was purchased from Frontier Scientific (Logan, UT, USA). Nanoparticles were formulated, manufactured using a high-pressure homogenizer, and characterized by dynamic light scattering and SEM as described for our previously reported procedure (8, 44).

### Assay of GaNP cytotoxicity.

The cytotoxic effects of GaNP on THP-1 macrophages were determined using a resazurin reduction assay. A total of 3 × 10^5^ THP-1 cells/well in a 48-well plate (0.75 × 10^6^ THP-1 cells/well in a 24-well plate) were placed and differentiated in the presence of phorbol myristate acetate (PMA; 7.5 ng/ml) for 24 h. The THP-1 macrophages were loaded with GaNP (25, 100, 300, 500 μM), washed after 24 h, and placed in culture with RPMI 1640 medium supplemented with 10% heat-inactivated fetal bovine serum (FBS). After 24 h or 15 days, the cells were washed thoroughly with phosphate-buffered saline (PBS; 5 times) and fresh medium was added. Forty microliters of a resazurin solution (0.15 mg/ml in Dulbecco's PBS; 100 μl for a 24-well plate) was added to each well. After incubation at 37°C for 3 h, fluorescence intensities were measured at a 560-nm excitation wavelength and a 590-nm emission wavelength.

### M. tuberculosis (H37Ra) and HIV-1 coinfection of human MDMs.

Human monocytes from healthy human donors, which were purchased from the UNMC Department of Pharmacology and Experimental Neuroscience cell core facility using an IRB-approved protocol, were prepared and purified by countercurrent centrifugal elutriation. All samples were provided in a deidentified fashion.

To induce differentiation into MDMs, the monocytes (0.75 × 10^6^ cells/well/ml in a 24-well tissue culture plate) were incubated in Dulbecco modified Eagle medium (DMEM) that was supplemented with 10% heat-inactivated pooled human serum (Innovative Biologics, Herndon, VA, USA), 50 μg/ml gentamicin (Mediatech Inc., Manassas, VA) to prevent the replication of extracellular bacteria, 10 ng/ml M-CSF (BioLegend, San Diego, CA), and 10 mM sodium pyruvate (Mediatech Inc., Manassas, VA) at 37°C in a 5% CO_2_ humidified atmosphere. Half the medium was replaced on the 5th day of incubation and then every 2 days thereafter until day 10 of incubation. At the 10th day, the differentiated MDMs were treated with DMEM supplemented with 1% human serum. On day 11, the MDMs were incubated with GaNP (300 μM) for 24 h at 37°C in a 5% CO_2_ humidified atmosphere. The drug-loaded MDMs were washed with PBS and incubated for up to an additional 15 days prior to infection to evaluate the long-acting potential of the Ga nanoparticles. Drug-treated MDMs were infected with M. tuberculosis (H37Ra) at the desired time points (1, 5, 10, or 15 days after drug loading) by incubating them for 4 h (multiplicity of infection [MOI] = 1) in medium lacking gentamicin. For studies of HIV infection only, macrophages were incubated with HIV-1_ADA_ (MOI = 0.01) for 24 h. For studies of HIV-1 and M. tuberculosis coinfection, the cells were incubated with HIV-1_ADA_ (MOI = 0.01) for 24 h after 4 h of infection with M. tuberculosis (MOI = 1) at days 1, 5, 10, and 15 following drug treatment (again, using medium without gentamicin). After infection, MDMs were washed with PBS to remove extracellular M. tuberculosis and/or HIV, and the cells were cultured in the same medium containing gentamicin described above to block the extracellular replication of M. tuberculosis.

### Determination of growth of M. tuberculosis bacteria residing in macrophages after treatment.

Drug-treated MDMs or control MDMs were infected with only M. tuberculosis, only HIV-1, or both M. tuberculosis and HIV-1 and lysed for analysis of the number of M. tuberculosis CFU in macrophages 2 days after infection(s). After the medium was removed from the wells, iced sterile water (300 μl) was added to the wells, followed by incubation on ice for 10 min. The MDMs were treated with 1.2 ml of lysis buffer containing 55% 7H9 broth, 20% 0.25% SDS, and 25% 20% bovine serum albumin in PBS. The lysed cells were centrifuged at 14,000 × *g* for 15 min, and the pellets were resuspended in 200 μl of PBS, serially diluted in sterile PBS, and plated onto 7H11 agar plates. The number of M. tuberculosis CFU was then counted after 3 weeks.

### M. tuberculosis (H37Ra) or HIV-1 growth inhibition in infected THP-1 macrophages by Ga nanoparticles.

THP-1 macrophages (0.75 × 10^6^ cells/well/ml in a 24-well tissue culture plate) were infected with either M. tuberculosis strain H37Ra (MOI = 1, with 4 h of incubation) or HIV-1 (MOI = 0.01, with 24 h of incubation) in RPMI 1640 medium that was supplemented with 10% heat-inactivated fetal bovine serum (no antibiotics were used). After washing with PBS buffer, the cells were treated with GaNP (300 μM) in medium (1% FBS) for 24 h. Extracellular nanoparticles were removed by washing the cells with PBS buffer three times. The treated cells were lysed at days 3 and 5 following infection to isolate H37Ra bacteria, and the growth of M. tuberculosis was monitored by determining the number of M. tuberculosis CFU as described above. For determination of HIV-1 growth, the medium was saved at days 4 and 5 and reverse transcriptase (RT) activity was assayed as described in the manufacturer's protocol (Enzchek, Molecular Probes, Eugene, OR, USA).

### Determination of growth of M. tuberculosis bacteria residing in THP-1 macrophages treated with Ga(NO_3_)_3_ or Fe(NO_3_)_3_.

THP-1 cells (7.5 × 10^5^ per well) were differentiated in RPMI 1640 medium containing 1% fetal serum, PMA (7.5 ng/ml), 10 mM sodium pyruvate, 50 μg/ml gentamicin, 0.75% NaHCO_3_, and 10 mM HEPES (pH 7.0) at 37°C in a 5% CO_2_ humidified atmosphere for 24 h. After washing with PBS buffer twice, the THP-1 cells were treated with 300 μM GaNP, Ga(NO_3_)_3_, or a combination of GaNP and Fe(NO_3_)_3_ in RPMI 1640 medium for 24 h. The growth of M. tuberculosis was determined as described above. In brief, drug-treated THP-1 macrophages were infected at the desired time points (5, 10, or 15 days after drug loading) with M. tuberculosis (H37Ra; MOI = 1) for 4 h in medium without gentamicin. In the case of combination treatment with GaNP and Fe(NO_3_)_3_, Fe(NO_3_)_3_ was continuously added to the culture medium until lysis for counting of the number of CFU.

### Determination of HIV growth in macrophages after infection.

For determination of the magnitude of HIV infection of MDMs, MDM-containing wells infected with only HIV-1 or both HIV-1 and M. tuberculosis (MOI = 1) were incubated for 11 days at 37°C, with the DMEM being replaced every 2 days. The cells were harvested at day 11 postinfection and stored at −80°C until RT activity determination. The RT assay (EnzChek) was performed as described in the manufacturer's protocol.

### Quantitation of cytokines.

Culture supernatants were analyzed using a multiplex kit from Life Technologies to assess the levels of human cytokines (granulocyte-macrophage colony-stimulating factor, IFN-γ, IL-1β, IL-2, IL-4, IL-5, IL-6, IL-8, IL-10, TNF-α) according to the manufacturer's instructions. In brief, the 96-well filter plate was prewetted, and then 25 μl of the diluted bead suspension was added to each well and the plate was washed twice. One hundred microliters of samples or standards was added to each well. The plate was incubated at room temperature for 2 h on a shaker. Following washing, biotinylated detection antibody was added and the mixture was incubated for 1 h. After washing, streptavidin–R-phycoerythrin was added to each well and the mixture was incubated for 0.5 h. The plate was again washed, resuspended in 100 μl of the washing buffer, and read on a Luminex xMAP system (AtheNA Multi-Lyte; Bio-Rad, USA). All samples were run in triplicate, and standards were run in duplicate.

### Whole-cell extracts and Western blot analysis.

MDMs cultured in 6-well plates were washed with ice-cold PBS buffer, and cell proteins were extracted in radioimmunoprecipitation assay buffer containing Halt protease inhibitor cocktail (Pierce Biotechnology, IL, USA) and Pierce phosphatase inhibitor (Pierce Biotechnology, IL, USA). The lysates were collected and centrifuged at 4°C at 12,000 × *g* for 10 min. The supernatants were collected, and a bicinchoninic acid assay was performed to determine the protein concentration. Protein samples were electrophoresed under denaturing conditions using 4 to 20% polyacrylamide gels (Bio-Rad, USA). After the membranes were transferred to a polyvinylidene difluoride membrane, the membranes were analyzed with goat polyclonal IKK-β antibody (Santa Cruz Biotechnology, CA, USA) and rabbit monoclonal NF-κB1 and phospho-Akt antibodies (Cell Signaling Technology, MA, USA). The bound antibody was detected using horseradish peroxidase-conjugated goat anti-rabbit IgG (Novex, MD, USA) or rabbit anti-goat IgG (Santa Cruz Biotechnology, CA, USA), and the membrane was developed with a chemiluminescent substrate (Pierce Biotechnology, IL, USA). ImageJ software was used for densitometric analysis of the membrane blots.

### Statistical analysis.

Statistical analysis was performed using Student's *t* test. Data are presented as the mean ± standard error of the mean. Data were considered significant at a *P* value of <0.05.

## Supplementary Material

Supplemental material
