# Supplementary material for: Ga(III) Nanoparticles Inhibit Growth of both Mycobacterium tuberculosis and HIV and Release of Interleukin-6 (IL-6) and IL-8 in Coinfected Macrophages
Source: Antimicrob Agents Chemother. 2017 Mar 24;61(4):e02505-16. doi: 10.1128/AAC.02505-16 (PMC5365726; doi:10.1128/AAC.02505-16)
Supplement: Supplemental material [file supp_61_4_e02505-16__index.html]

Supplemental material 

# Ga(III) Nanoparticles Inhibit Growth of both Mycobacterium tuberculosis and HIV and Release of Interleukin-6 (IL-6) and IL-8 in Coinfected Macrophages

## Supplemental material

- Supplemental file 1 -

  Supplemental Figures S1 to S11

  PDF, 466K
