# Supplementary material for: Ga(III) Nanoparticles Inhibit Growth of both Mycobacterium tuberculosis and HIV and Release of Interleukin-6 (IL-6) and IL-8 in Coinfected Macrophages
Source: Antimicrob Agents Chemother. 2017 Mar 24;61(4):e02505-16. doi: 10.1128/AAC.02505-16 (PMC5365726; doi:10.1128/AAC.02505-16)
Supplement: Supplemental material [file AAC.02505-16_zac004176069s1.pdf]

Supporting Information

Ga(III) Nanoparticles Inhibit Growth of Both TB and HIV and Release of IL-6 and IL-8 in Co-Infected Macrophages

Seoung-ryoung Choi[1], *Bradley E. Britigan*[1,2,3] *and Prabagaran Narayanasamy*[1,*]

[1]Department of Pathology and Microbiology, [2]Department of Internal Medicine, College of Medicine, University of Nebraska Medical Center, Omaha, Nebraska 68198 and [3]Research Service, VA Medical Center-Nebraska Western Iowa, Omaha, Nebraska 68105.

Corresponding author: p.narayanasamy@unmc.edu

Figure **S1**. Toxicity of Ga nanoparticle (GaNP) against THP-1 macrophages……………………………..2

Figure **S2**. Antimicrobial activities of GaNP and combination of GaNP and $FeNO_3$…………………….2

Figure **S3**. *M.tb* (H37Ra) growth inhibition in THP-1 macrophages by Ga nanoparticles or $GaNO_3$…….2

Figure **S4**. Fluorescence intensity of IL-6 and IL-8 in supernatants from TB-infected macrophages….…..3

Figure **S5**. Fluorescence intensity of IL-1 presented in supernatants from co-infected macrophages……..3

Figure **S6**. Fluorescence intensity of TNF-α presented in supernatants from co-infected macrophages…..3

Figure **S7**. Fluorescence intensity of IL-4 presented in supernatants from co-infected macrophages……..4

Figure **S8**. Fluorescence intensity of GMCSF presented in supernatants from co-infected macrophages…4

Figure **S9**. Fluorescence intensity of IFN-γ presented in supernatants from co-infected macrophages…...4

Figure **S10**. Release of IL-6 and IL-8 by MDMs in the presence of MCSF……………………………..…5

Figure **S11**.Western blot analysis of pP38 and pAkt from macrophages treated with GaNP………………5

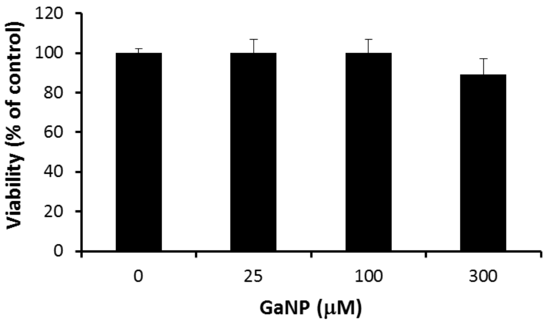

Figure **S1**. Toxicity of Ga nanoparticle (GaNP) against THP-1 macrophages. In a 48 well plate, 3 × 10^5 cells/well were seeded. The wells were treated with various concentrations of GaNP for 24 hour, and then the wells were washed with PBS buffer thoroughly. The GaNP-treated macrophages were further incubated for additional 24 hour and viability was determined using resazurin reduction assay.

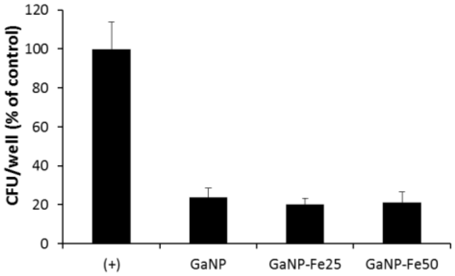

Figure **S2**. Antimicrobial activities of GaNP and combination of GaNP and $FeNO_3$. THP-1 macrophages (0.67 × 10^6) were incubated with GaNP (300 µM) or combination of GaNP (300 µM) and $FeNO_3$ (25 or 50 µM) for 24 hours, following which the cultures were washed free of extracellular drug. These macrophages were then infected with *M.tb* (H37Ra, MOI = 1) for 4 hours and allowed to grow for 2 days. Data represents the mean ± SEM of triplicate (n = 3). (+): *M.tb* Infection.

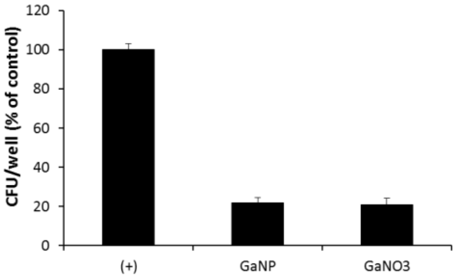

Figure **S3**. *M.tb* (H37Ra) growth inhibition in THP-1 macrophages by Ga nanoparticles or $GaNO_3$. THP-1 macrophages (0.67 × 10^6) were incubated with GaNP (300 µM) or and $GaNO_3$ (300 µM) for 24 hours. The cultures were washed free of extracellular drug with PBS buffer. These macrophages were then infected with *M.tb* (H37Ra, MOI = 1) for 4 hours and allowed to grow for 2 days at day 5 following treatments. Growth of *M.tb* was monitored over time by determining *M.tb* CFU as per Methods. Data represents the mean ± SEM of triplicate (n = 3). (+): *M.tb* Infection.

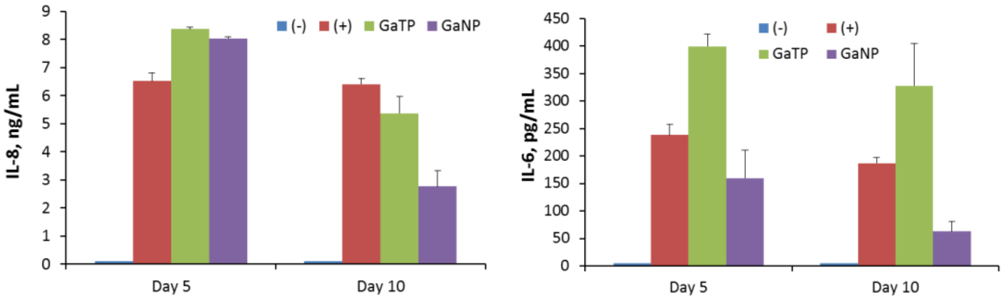

Figure S4. Fluorescence intensity of IL-6 and IL-8 presented in supernatants from TB-infected macrophages. MDMs were co-infected with *M.tb* (H37Ra) at Day **5** and **10** following drug treatment. Following infections, supernatants were analyzed for detection of cytokines released from infected macrophages day 2 after infection. (+); positive control (infection only). (-); negative control (no infection).

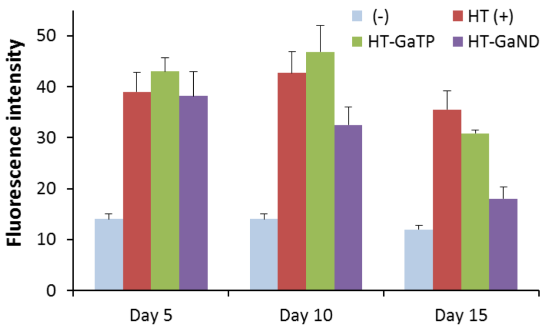

Figure S5. Fluorescence intensity of IL-1 presented in supernatants from co-infected macrophages. MDMs were co-infected with HIV and *M.tb* (H37Ra) at Day **5, 10** and **15** following drug treatment. Following infections, supernatants were analyzed for detection of cytokines released from infected macrophages day 2 after infection. HT; HIV/TB co-infection. (+); positive control. (-); negative control.

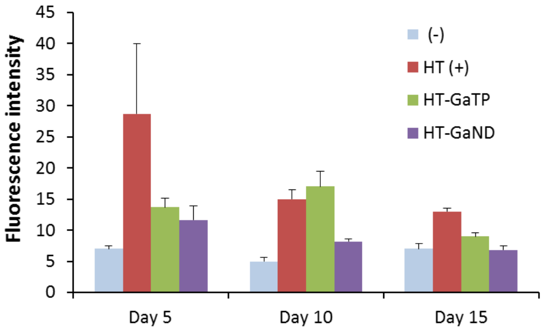

Figure S6. Fluorescence intensity of TNF-α presented in supernatants from co-infected macrophages. MDMs were co-infected with HIV and *M.tb* (H37Ra) at Day **5, 10** and **15** following drug treatment. Following infections, supernatants were analyzed for detection of cytokines released from infected macrophages day 2 after infection. HT; HIV/TB co-infection. (+); positive control. (-); negative control.

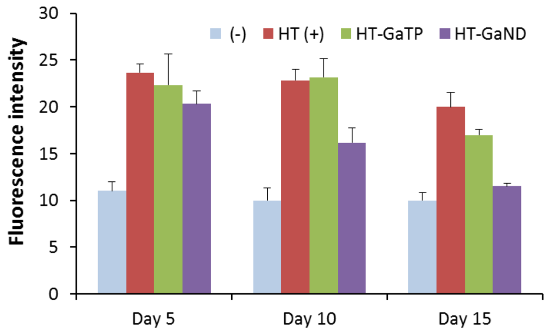

Figure S7. Fluorescence intensity of IL-4 presented in supernatants from co-infected macrophages. MDMs were co-infected with HIV and *M.tb* (H37Ra) at Day **5, 10** and **15** following drug treatment. Following infections, supernatants were analyzed for detection of cytokines released from infected macrophages day 2 after infection. HT; HIV/TB co-infection. (+); positive control. (-); negative control.

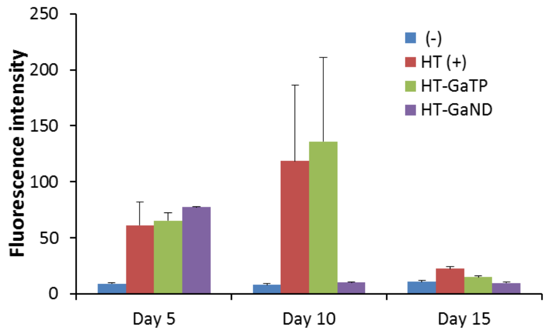

Figure S8. Fluorescence intensity of GMCSF presented in supernatants from co-infected macrophages. MDMs were co-infected with HIV and *M.tb* (H37Ra) at Day **5, 10** and **15** following drug treatment. Following infections, supernatants were analyzed for detection of cytokines released from infected macrophages day 2 after infection. HT; HIV/TB co-infection. (+); positive control. (-); negative control.

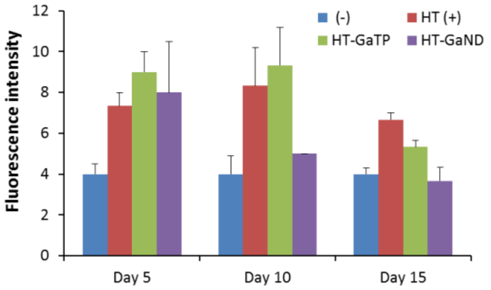

Figure S9. Fluorescence intensity of IFN-γ presented in supernatants from co-infected macrophages. MDMs were co-infected with HIV and *M.tb* (H37Ra) at Day **5, 10** and **15** following drug treatment. Following infections, supernatants were analyzed for detection of cytokines released from infected macrophages day 2 after infection. HT; HIV/TB co-infection. (+); positive control. (-); negative control.

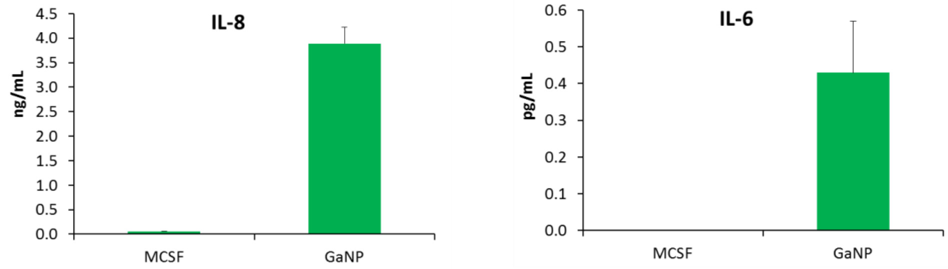

Figure S10. Release of IL-6 and IL-8 by MDMs in the presence of MCSF, compared with GaNP treated MDMs exposed to the same agents.

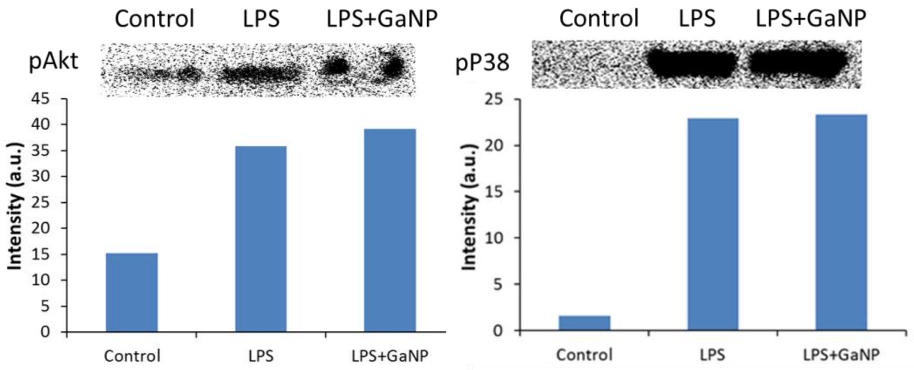

Figure S11.Western blot analysis of pP38 and pAkt from macrophages treated with GaNP. Macrophages were polarized by LPS. No changes were observed in expressions of pP38 and pAkt between GaNP-treated and non-treated macrophages . Control: No polarization of monocytes by LPS.
